# Evaluating comparative effectiveness of pembrolizumab-based therapy versus chemotherapy in treatment of gastric carcinoma: a systematic review and meta-analysis of randomized controlled trials

**DOI:** 10.1007/s10238-025-01610-5

**Published:** 2025-03-28

**Authors:** Haleema Mansoor, Maheen Gohar, Asma Attaria, Faiza Fatima Karim, Umaimah Naeem, Mohsin Khan, Javed Iqbal

**Affiliations:** 1 Jinnah Sindh Medical University, Karachi, Pakistan; 2 Liaquat University of Health Sciences, Jamshoro, Pakistan; 3 Nursing Department, Communicable Disease Center, Hamad Medical Corporation, P.O Box 3050, Doha, Qatar

**Keywords:** Pembrolizumab, Gastric carcinoma, Chemotherapy

## Abstract

Gastric cancer, especially cancer of the gastro-esophageal junction, ranks among the first five cancers in the world with the highest mortality rates. It has poor survival rates for the advanced stages. Traditional chemotherapy, while standard, often results in significant side effects and limited efficacy. The objective of this meta-analysis and systemic review is to ascertain if pembrolizumab-based therapies for advanced gastric cancer are more effective and safer than standard chemotherapy. The focus consisted of RCTs with adults suffering from gastric carcinoma who received pembrolizumab every 3 weeks (200 mg) intra-related dose or with at least comparable chemotherapy regimen. Outcomes assessed are as follows: overall survival (OS), progression-free survival (PFS), and objective response rate (ORR). All potential sources regarding the search of outcome measures were applied: Google Scholar, Scopus, PubMed, and Cochrane library, and last search in June 2024 was carried out. Out of 568 articles screened, four RCTs comprising 2,831 patients met the inclusion criteria. Analysis indicated that pembrolizumab alone did not significantly improve OS compared to chemotherapy (HR 0.87). However, when combined with chemotherapy, pembrolizumab dramatically enhanced OS (HR 0.80) and PFS (HR 0.78). ORR was superior in the pembrolizumab plus chemotherapy group (RR 1.24), while pembrolizumab monotherapy showed no significant difference from chemotherapy alone. Safety analysis revealed a higher frequency of adverse events in the pembrolizumab-based therapy groups compared to chemotherapy. Pembrolizumab together with chemotherapy improves greater survival and higher levels of response rate in patients with severe gastric cancer, especially with high PD-L1 expression. But it has rather more adverse events, allowing patient monitoring with care.

## Introduction

Gastric cancer, particularly gastro-esophageal junction (GEJ) cancer, is a malignant disease that begins in the stomach’s lining. It is regarded as the fifth most common cancer diagnosed worldwide and the third most common cause of cancer-related deaths. More than a million new cases were recorded in 2018 alone, and the illness accounted for nearly 783,000 casualties [1]. There are geographical variations in stomach cancer rates, with Eastern Asia—especially South Korea, Japan, and China—having the highest rates [2]. The higher prevalence observed in these regions is influenced by a variety of variables, including eating habits, inherited attributes, and Helicobacter pylori infection [2]. Despite treatment options such as radiation, chemotherapy, and surgery, patients with advanced stomach cancer have a poor prognosis, with less than 10% projected to survive for five years [3].

Fluoropyrimidine and platinum-based drugs, which hinder cancer cells from DNA replication and decrease the disease’s progression while relieving symptoms, are often employed in chemotherapy treatments [1]. Patients who undergo chemotherapy solely usually have a median survival of under one year, and its adverse effects may significantly decrease their quality of life [1]. The adverse effects of chemotherapy, which include bone marrow suppression, neuropathy, nausea, and vomiting, limit its efficacy as a long-term treatment for advanced stomach cancer and raise the risk of infection [1]. A monoclonal antibody called pembrolizumab, which targets the PD-1 receptor, has demonstrated promise as an alternative medical treatment, specifically for individuals whose tumors are PD-L1 positive [4]. Pembrolizumab inhibits the linkage of PD-1 on T cells to its ligands, PD-L1 and PD-L2, both of which are usually excessively expressed in tumor cells and throughout the tumor microenvironment [4]. T cells that would normally be immune system-tolerable are reactivated by this suppression, which enhances their capacity to identify and eliminate cancer cells [4]. When paired with additional chemotherapy drugs, pembrolizumab has been notably beneficial for individuals with advanced gastric cancer [4]. Pembrolizumab showed a strong objective response in patients with metastatic or recurrent gastric cancer in the KEYNOTE-059 study, especially those whose PD-L1 combined positive score (CPS) was 1 or higher [5]. KEYNOTE-061 [4] and KEYNOTE-0621 are two trials that have assessed the usage of pembrolizumab both alone and in combination with chemotherapy [4]. According to these studies, pembrolizumab has fewer adverse effects than conventional chemotherapy and, in some circumstances, can result in long-lasting outcomes, particularly in individuals who have elevated PD-L1 expression. [4]. Compared to standard chemotherapy, pembrolizumab is less toxic and may be more successful in prolonging survival for some patients because of its function as an immune checkpoint inhibitor [3]. PD-L1 levels and tumor mutational burden are two variables that affect patient response; therefore, its effectiveness is not consistent across the board [1, 4].

The purpose of this systematic review and meta-analysis is to compare chemotherapy with pembrolizumab-based treatments for advanced gastric cancer. It is crucial to investigate novel treatments that can enhance patient outcomes because traditional chemotherapy has substantial side effects and a limited efficacy [3]. Pembrolizumab has shown encouraging results in clinical trials, which could raise the overall survival rate and quality of life, particularly for patients with elevated PD-L1 expression [4]. As suggested by the findings of studies like KEYNOTE-059 and KEYNOTE-061, where pembrolizumab demonstrated efficacy and a strong safety profile in particular patient groups, data from randomized controlled trials will be combined in this investigation to assess important outcomes such as rates of responses, progression-free survival, and the overall survival rate. This meta-analysis’s conclusions may have a substantial influence on clinical procedures and future approaches to treating advanced gastric cancer [4, 5].

### Methodology

The protocol of this systemic review and meta-analysis was registered in the PROSPERO International Prospective Register of Systematic Reviews (registration No. CRD42024572775) available at https://www.crd.york.ac.uk/prospero/display_record.php?ID=CRD42024572775.

#### Eligibility criteria

Randomized controlled trials, also known as cluster RCTs, that compare a pharmacological intervention (pembrolizumab-based therapy) with another pharmacological intervention (chemotherapy alone) for the treatment of gastric cancer are the only ones we will consider.

The **inclusion criteria** are:

**Participants:** Adult patients aged 18 and up who have been diagnosed with gastric carcinoma, regardless of tumor stage or previous treatment history. No restriction on race, sex, place, ethnicity, or language.

**Intervention:** Pharmacological intervention: Pembrolizumab (Anti-PD-1 therapy).

Standard dose: 200 mg every 3 week for one year or until the illness returns or the toxicity becomes intolerable.

**Comparator:** Any type of chemotherapy.

**Primary Outcomes:** The following are the main outcomes of the study:

1. Comparison of overall survival (OS) of pembrolizumab-based therapy vs chemotherapy.

2. Comparison of progression-free survival (PFS) of pembrolizumab-based therapy vs chemotherapy.

3. Comparison of the objective response rate (ORR) in the two groups.

#### Additional outcome(s)

1- Any grade negative occurrences.

2- Adverse occurrences of grades 3–5.

3- Termination of treatment.

### Exclusion criteria

We will not include studies that provide reviews of trials that will be published independently, or uncontrolled trials. All observational research, including case control, will be disregarded, retrospective or prospective cohort, and cross-sectional studies. Peer review articles, commentaries, letter to editors, and case reports would be excluded.

It includes uncontrolled trials; Cochrane reviews; non-randomized studies; or trials with fewer than ten participants in each group.

#### Information sources

The following databases were searched: Google Scholar [6], Scopus [7], PubMed [8] as well as the Cochrane Library [9]. Additional clinical studies were obtained from Clinical Trials. gov. June 2024 was the last research. The included studies were also manually screened from the reference lists for additional relevant studies.

#### Search strategy

The following terms were part of the search strategy:

We used following Medical Subject Headings terms (MESH) and keywords:

((“Stomach Neoplasms”[Mesh]) AND (Pembrolizumab OR “pembrolizumab” [Supplementary Concept]) AND (“Chemotherapy, Adjuvant”[Mesh]) AND (“Disease-Free Survival” OR “Disease-Free Survival”[Mesh]) AND (RCT OR Randomized Controlled Trials OR Randomized Controlled Trials).

No restrictions regarding race, place, sex, ethnicity, language, or dates were applied.

#### Selection process

Two independent reviewers examined each selected study, and the abstract and title. We assessed full texts of studies with potentially relevant study designs based on defined eligibility criteria. In case of disagreements, we discussed or consulted with a third reviewer in order to resolve them.

#### Data collection process

Using a standardized form, two reviewers separately extracted data. Extracted data included:

- Author, year, and country.

- Age, gender, and disease duration of the participant.

- Overall survival rate and pembrolizumab progression-free survival plus chemotherapy therapy vs chemotherapy alone.

- Tumor PD-L1 expression and safety data.

A third reviewer resolved the inconsistencies in the data. Where missing information was needed, we requested study authors to provide additional information.

#### Data items

##### Primary outcomes

*Overall survival (OS)*

When comparing individuals receiving pembrolizumab alone or in combination with chemotherapy to those receiving chemotherapy alone, OS shows the hazard ratio (HR) and 95% confidence interval (CI), which indicate the chance of survival. A lower HR suggests improved survival, with significance assessed by p-values.

*Progression-free survival (PFS)*

PFS calculates the proportion of patients remaining free from disease progression, comparing pembrolizumab monotherapy or combination therapy with chemotherapy. Lower HR values indicate longer progression-free periods, with statistical significance confirmed by p-values.

*Overall response rate (ORR)*

ORR is the proportion of patients who achieve a predefined tumor reduction, comparing pembrolizumab alone, with chemotherapy, and chemotherapy alone. Results are expressed as relative risk (RR) with a 95% CI, where an RR above 1 indicates higher response rates, with p-values showing statistical significance.

**Secondary outcomes**: Safety profiles and adverse effects.

#### Risk of bias assessment

Every included study was evaluated for bias risk using the Cochrane Collaboration’s Risk of Bias Tool. Two separate reviewers examined the studies, and they discussed or referred any disagreements to a third reviewer.

#### Effect measures

Risk ratios (RRs) with 95% CIs were calculated for dichotomous outcomes (ORR). To estimate survival times, continuous outcomes (OS and PFS) were evaluated using hazard ratios (HRs) with 95% CIs.

#### Synthesis methods

We conducted a meta-analysis on the outcome data since the comparators and study designs were sufficiently homogenous. We utilized random effect meta-analysis in Revman to account for the predicted heterogeneity among the included papers. The random effect model assumes that different studies estimate distinct but related intervention effects.

Heterogeneity between studies was accounted for through a random effect meta-analysis. A narrative synthesis was offered if studies were too heterogeneous. *I*^2^ to evaluate the heterogeneity, a statistic was employed. Participant characteristics and type of outcome were used as subgroup analyses. Excluding high risk studies was refrained sensitivity analyses.

Revman (V.5.1) [10] was used to compile and statistically analyze the effectiveness data. The relative risk with 95% confidence intervals (Cis) was used to calculate dichotomous data, and the weighted mean difference (WMD) or standardized mean difference (SMD) with 95% confidence intervals was used to evaluate continuous data. While SMD was utilized for different instruments, WMD was used for the same scale or evaluation tools. The omnibus homogeneity test (Q) was used to evaluate the heterogeneity (1) using the following metrics: There are four levels of heterogeneity, such as not significant (0%–40%), moderate (30–60%). substantial (50–90%), and significant (75–100%).

#### Reporting bias assessment

We used the Cochrane Collaboration Risk of Bias to evaluate each study’s risk of Bias Tool [11]. These decisions were based on the criteria for assessing the risk of bias and were decided by two independent review authors. The dispute was resolved by discussion first, followed by consultation and arbitration by the third author.

#### Certainty assessment

The overall certainty of the evidence for each outcome was evaluated using the GRADE (Grading of Recommendations, Assessment, Development and Evaluation) approach, which takes into account publication bias, risk of bias, consistency, directness, and accuracy.

This manuscript is reported in accordance with PRISMA guidelines for systematic reviews and meta-analysis [12].

## Results

### Study selection and characteristics

**Fig. 1:**
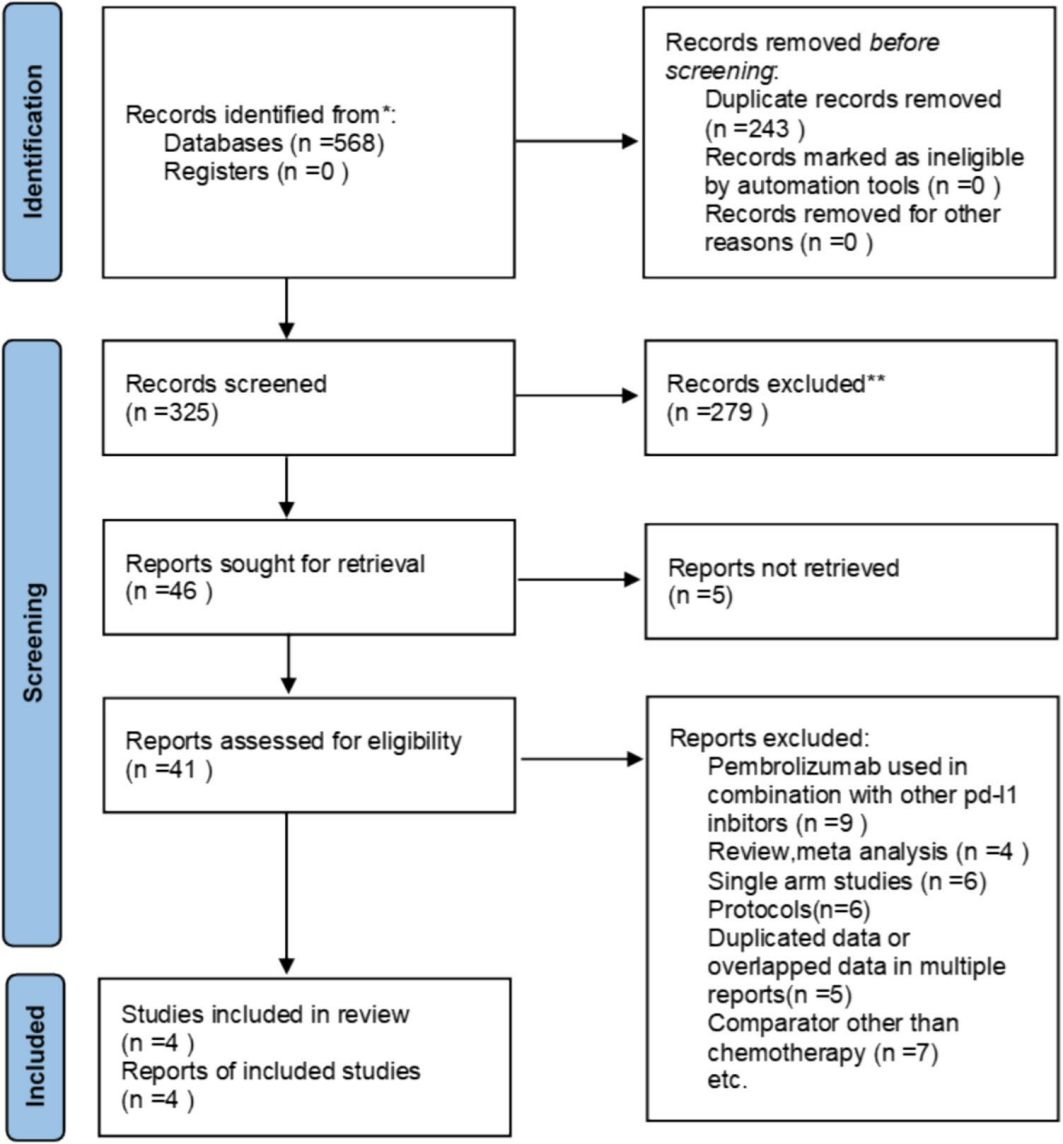
shows the flow diagram for the Preferred Reporting Items for Systematic Reviews and Meta-Analyses (PRISMA)

**Fig. 2:**
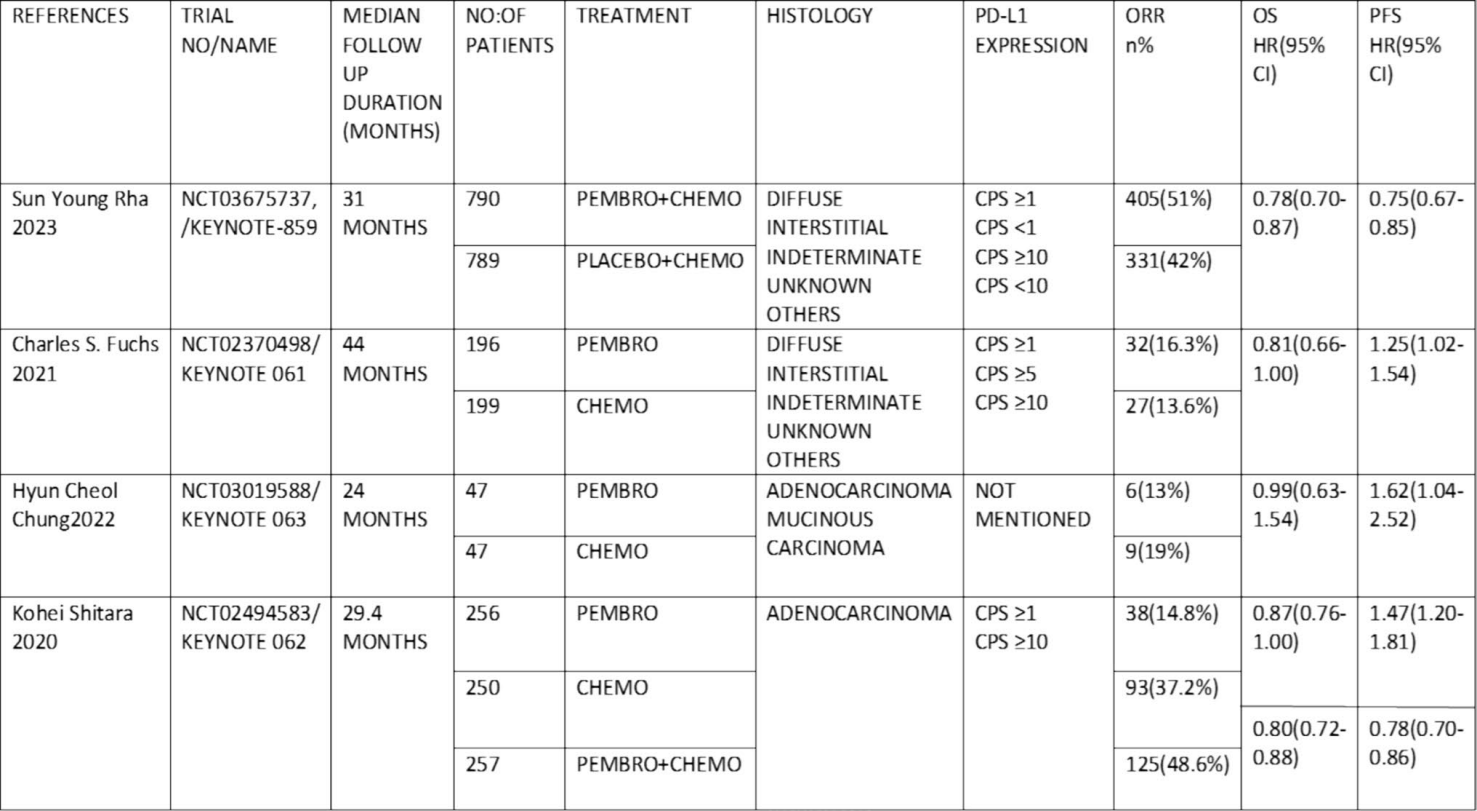
Characteristics of included studies

The original search resulted in 568 items. Figure 1 shows the filtering process, which rejected 564 papers and selected 4 RCTs [1, 2, 4, 13] with a total of 2,831 patients for meta-analysis. The median follow-up duration was between 24 and 31 months. For up to two years, the intervention group received 200 mg of pembrolizumab every three weeks, either with or without chemotherapy, whereas the control group only received chemotherapy. All studies reported PD-L1 expression as a CPS score, which was calculated by dividing the number of PD-L1-staining cells (tumor cells, macrophages, and lymphocytes) by the total number of viable tumor cells and multiplying by 100. Figure 2 shows main characteristics and outcomes of individual studies.

**Fig. 3:**
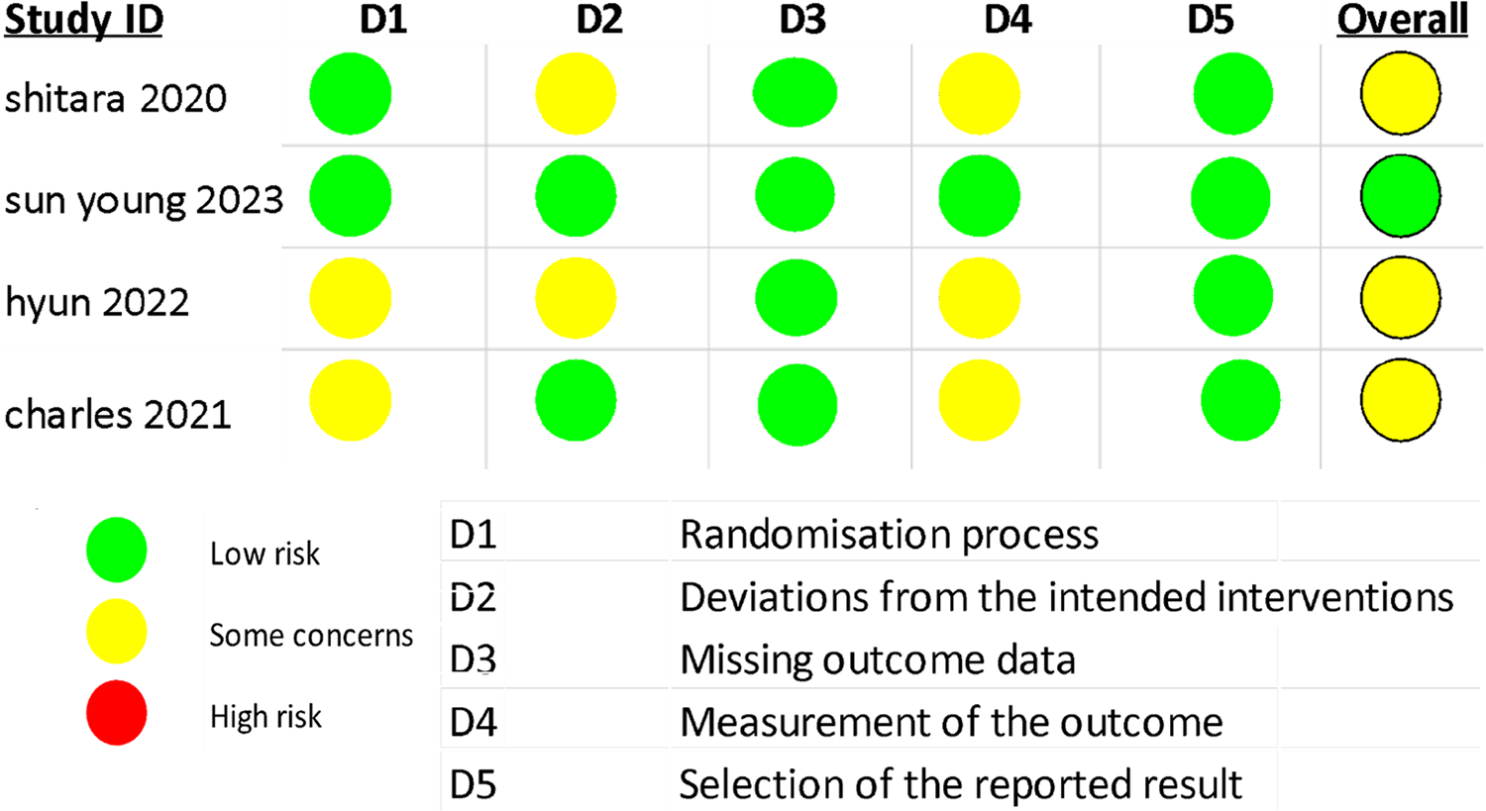
Risk of bias assement of included studies

#### Quality assessment

Every included study was evaluated for bias risk using the Cochrane Collaboration’s Risk of Bias Tool. Figure 3 displays the quality assessment results. Low-to-moderate risk of bias was reported by all RCTs. One of the trials was double blind, and the other three were open label.

**Fig. 4:**
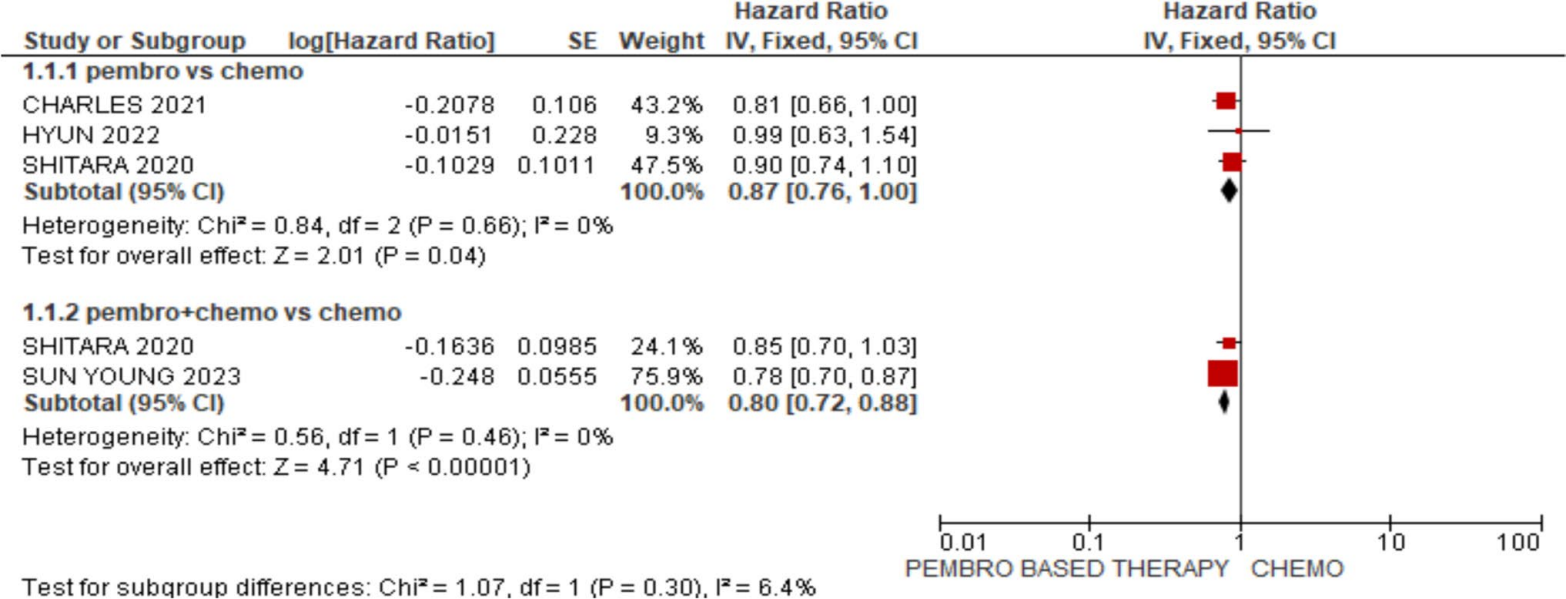
Forest plot for OS

#### Overall survival (OS)

Pembrolizumab by itself did not show any improvement in OS, according to our study [HR 0.87; 95% CI (0.76, 1.00), *p* = 0.04]. However, compared to patients receiving chemotherapy alone, those receiving pembrolizumab with chemotherapy had a significantly better overall survival rate [HR 0.80; 95% CI (0.72, 0.88), *p* < 0.00001], as shown in Fig. 4.

### Progression-free survival (PFS)

**Fig. 5:**
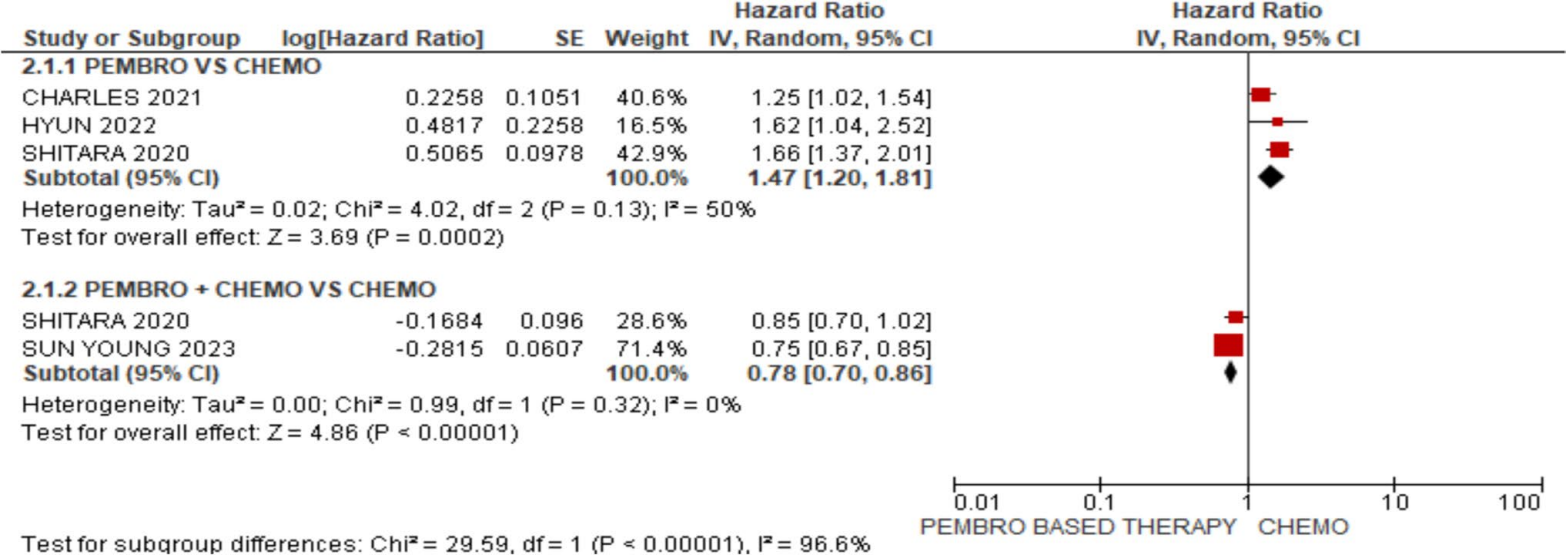
Forest plot for PFS

In terms of PFS, chemotherapy was found to be superior to pembrolizumab, [HR 1.47; 95% CI (1.20, 1.81), *p* = 0.0002] but pembrolizumab in addition to chemotherapy had a significant advantage over chemotherapy, [HR 0.78; 95% CI (0.70, 0.86), *p*, 0.00001], as shown in Fig. 5.

### Objective response rate (ORR)

**Fig. 6:**
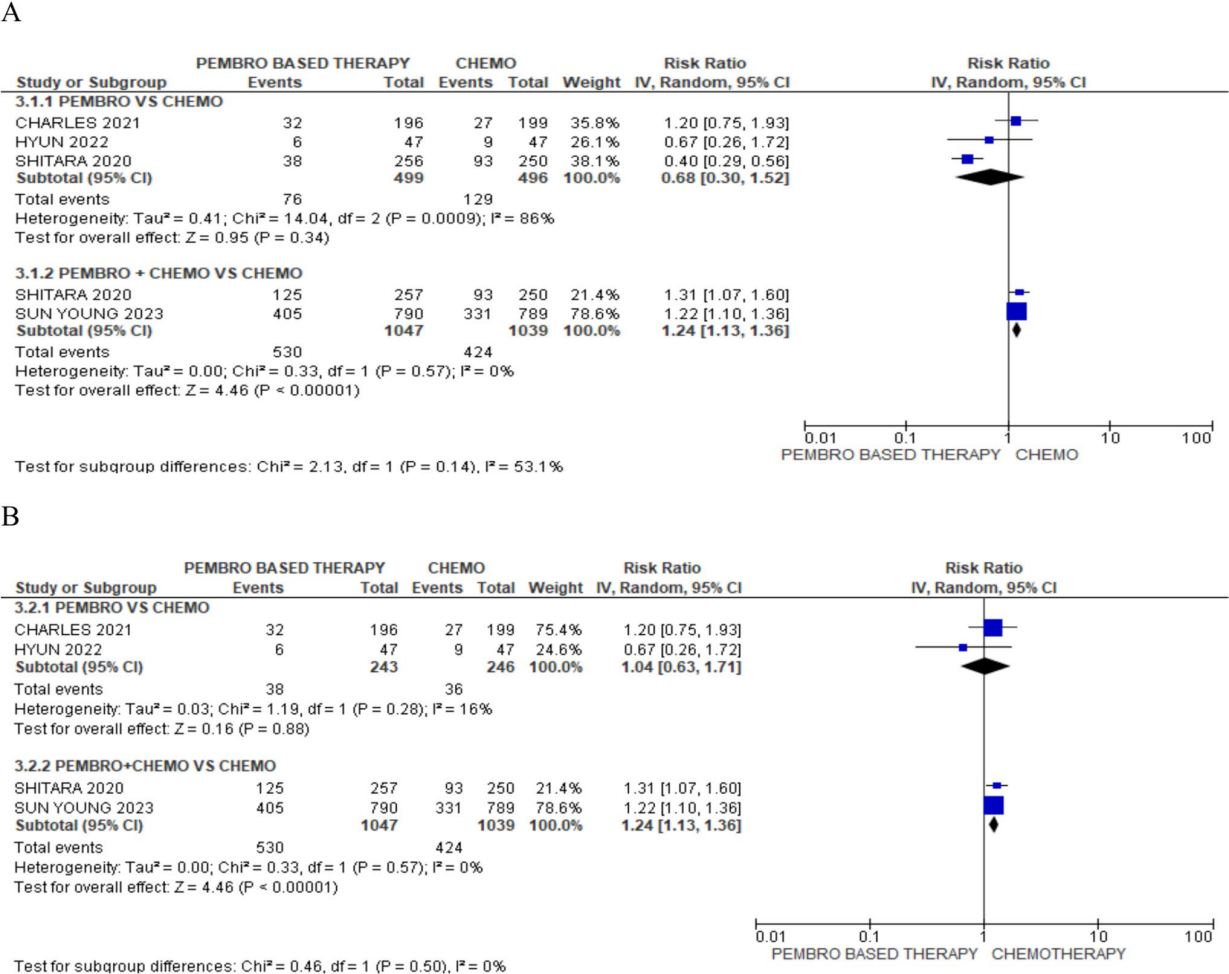
Forest plot of ORR. A Forest plots of ORR for pembrolizumab alone or in combination with chemotherapy; CI, confidence interval; B Forest plots of ORR for pembrolizumab alone or in combination with chemotherapy after removing one study; CI, confidence interval

For ORR, pembrolizumab monotherapy did not show statistically significant difference as compared with chemotherapy, [RR: 0.68; 95% CI (0.30, 1.52), *p* = 0.34]. However, pembrolizumab and chemotherapy synergy showed better ORR compared with chemotherapy alone, [RR 1.24; 95% CI (1.13, 1.36), *p* < 0.00001]. Heterogeneity of the pembrolizumab monotherapy group was found to be *I*^2^ = 86%. Sensitivity analysis was performed which suggested that SHITARA (2020) [1] may be the source of heterogeneity. After excluding SHITARA (2020), acceptable heterogeneity was obtained as *I*^2^ = 16%, [HR 1.04; 95% CI (0.63, 1.71), *p* = 0.88], as evident in Fig. 6.

## Safety

**Fig. 7:**
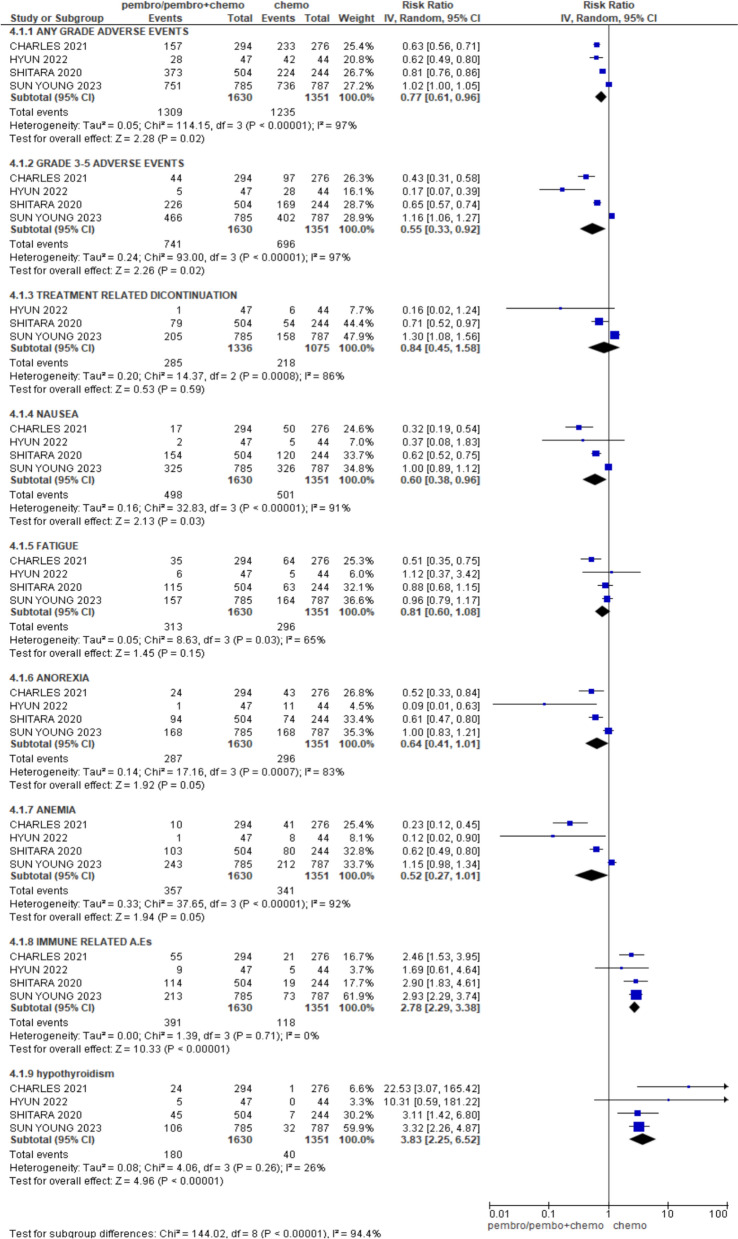
Forest plot for adverse events and safety

Pembrolizumab-based therapy (pembro/pembro + chemo) was associated with increased rate of adverse events of any grade and grade 3–5 in comparison with chemotherapy alone, [HR 0.77; 95% CI (0.61, 0.96), *p* = 0.02], [HR 0.55; 95% CI (0.33, 0.92), *p* = 0.02]. No significant difference (between pembrolizumab-based therapy and chemotherapy) was obtained regarding treatment-related discontinuation, fatigue, anorexia, and anemia, [HR 0.84; 95% CI (0.45, 1.58), *p* = 0.59], [HR 0.81; 95% CI (0.60, 1.08), *p* = 0.15], [HR 0.64; 95% CI (0.41, 1.01), *p* = 0.05], [HR 0.52; 95% CI (0.27, 1.01), *p* = 0.05]. However, incidence of nausea was greater in pembrolizumab-based therapy group as compared to chemotherapy group, [HR 0.60; 95% CI (0.38, 0.96), *p* = 0.03] (Fig. 7).

Compared with chemotherapy, pembrolizumab-based therapy exhibited a statistical significance in immune-related adverse effects (IRAE), [HR 2.78; 95% CI (2.29, 3.38), *p* < 0.00001]. The risk of hypothyroidism (an IRAE) was statistically higher for pembrolizumab-based therapy [HR 3.83; 95% CI (2.25, 6.52), *p* < 0.00001].

### Subgroup analysis on basis of tumor PD-L1 expression

Population with PD-L1 expression of CPS < 1 did not show any OS benefit from pembrolizumab combination therapy [HR 0.92; 95% CI (0.72, 1.17), *p* = 0.51], and there was no relevant data based on pembrolizumab monotherapy for this subgroup. Pembrolizumab-based combination therapy improved overall survival for patients with PD-L1 CPS ≥ 1 [HR 0.77; 95% CI (0.68, 0.89), *p* = 0.002], while monotherapy did not improve overall survival for those who received pembrolizumab-based monotherapy [HR 00.84; 95% CI (0.72, 1.00), *p* = 0.04]. However, population with PD-L1 CPS ≥ 10 has significantly enhanced OS with pembrolizumab monotherapy as well as with combination therapy (pembro + chemo) ([HR 0.69; 95% CI (0.53, 0.90), *p* = 0.006] and [HR 0.71; 95% CI (0.54, 0.95), *p* = 0.02]).

**Table:** 

PD-L1 CPS score	Sub group	No: of studies	Test of association			Test of heterogenity	
			HR	CL 95%	*P* value	*I*^2^	*P* value
PD-L1 CPS < 1	Monotherapy	–	–	–	–	–	–
	Combination therapy	1	0.92	0.73–1.17	0.51	–	–
PD-L1 CPS ≥ 1	Monotherapy	2	0.84	0.72–1.00	0.04	0%	0.54
	Combination therapy	2	0.77	0.68–0.89	0.002	35%	0.21
PD-L1 CPS ≥ 10	Monotherapy	2	0.69	0.53–0.90	0.006	0%	0.98
	Combination therapy	2	0.71	0.54–0.95	0.02	59%	0.12

#### Publication bias

**Fig. 8:**
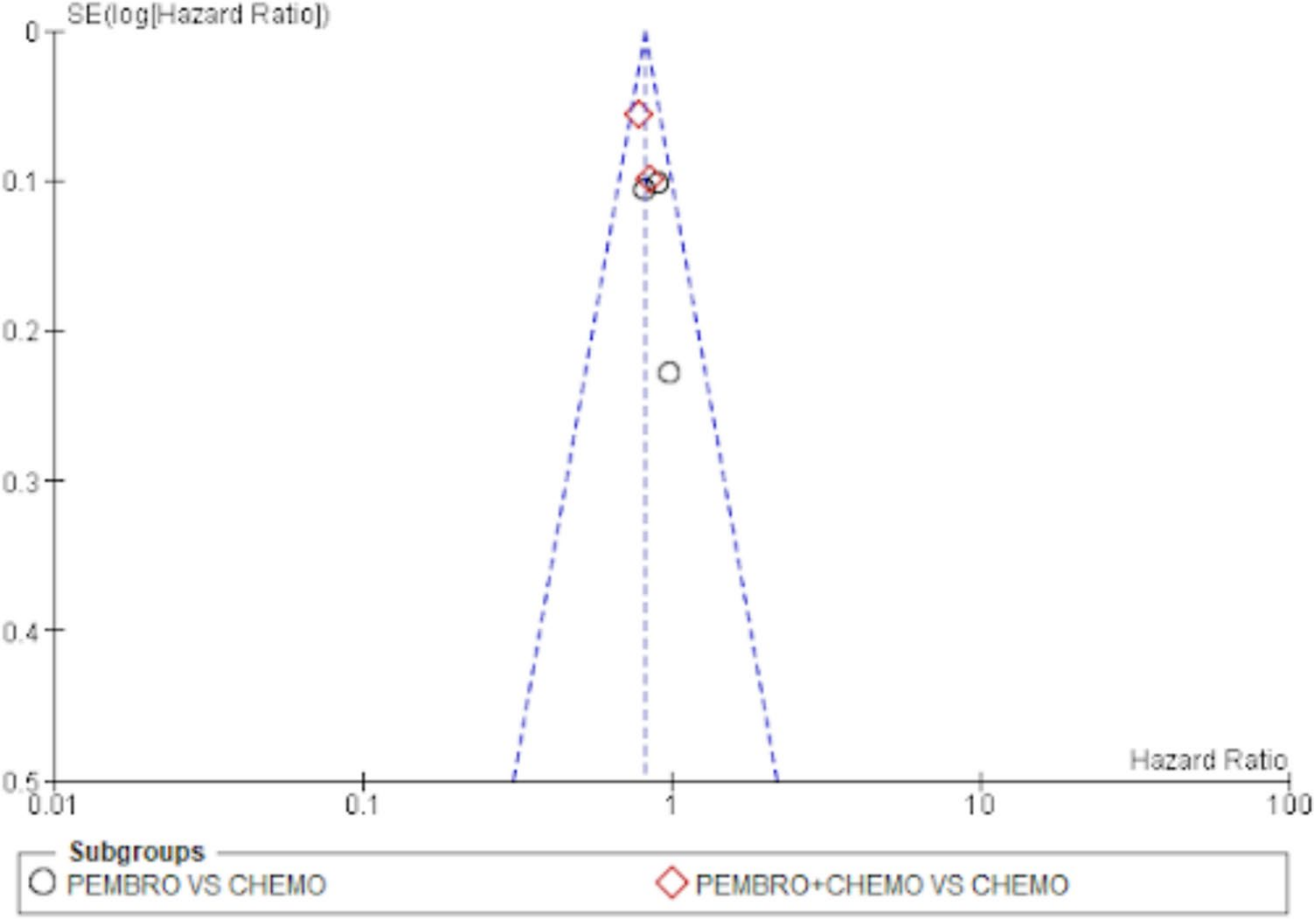
Funnel plot for overall survival

**Fig. 9:**
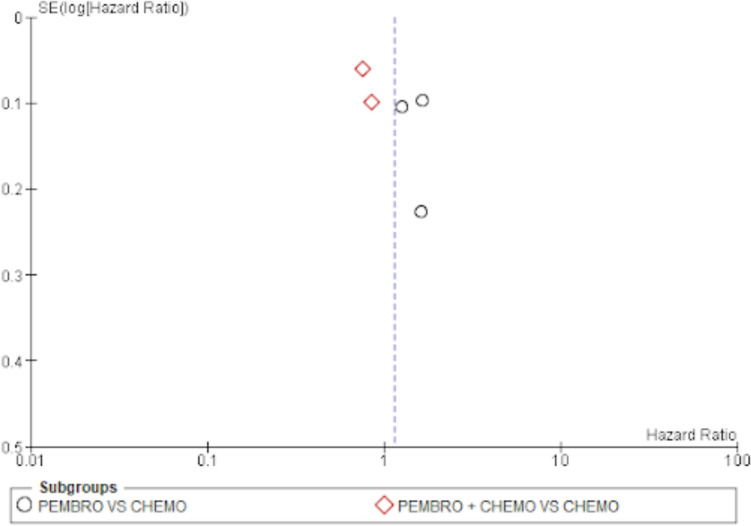
Funnel plot for progression-free survival

**Fig. 10:**
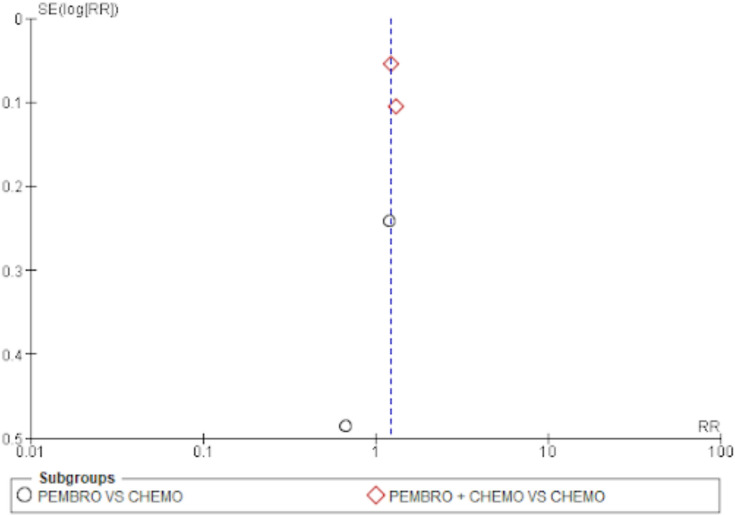
Funnel plot for objective response rate

The evaluation of funnel plots was done by looking at them. There appears to be no publishing bias, as indicated by the overall survival funnel plot’s symmetrical funnel shape and evenly distributed points. The same is true for funnel plots of the objective response rate and progression-free survival rate (Figs. 8, 9 and 10).

## Discussion

PD-1, an inhibitor of both adaptive and innate immune responses, is expressed by activated T cells, natural killer (NK) and B cells, macrophages, dendritic cells (DCs), and monocytes [14]. Interestingly, T lymphocytes specific to tumors have high levels of PD-1 expression [15]. An essential immune checkpoint protein, PD-1, interacts with PD-L1 (B7-H1) and PD-L2 (B7-H2) in that order. Antigen-presenting cells express PD-L2, while immune and epithelial cells inductively express PD-L1. To prevent immune system dysregulation, PD-1 physiologically interacts with antigen-presenting cell surface PD-L1 and PD-L2 [16]. Tumor cells that overexpress PD-L1 promote PD-1 binding to PD-L1 molecules on their surface, which hinders T cell immune surveillance, makes it more difficult to identify and eliminate tumor cells, and promotes tumor immune escape [17]. By disrupting PD-1/PD-L1 interactions, PD-1/PD-L1 monoclonal antibodies can be used to eradicate tumors, kill tumor-specific T cells again, and break tumor immune tolerance [18]. Pembrolizumab, a PD-L1 inhibitor, had encouraging efficacy with manageable safety in those with advanced stomach or gastro-esophageal junction cancer who had received at least two lines of treatment. Patients with metastatic or recurrent gastric cancer who had a PD-L1 combined positive score (CPS) of 1 or higher showed a significant objective response to pembrolizumab in the KEYNOTE-059 trial. Numerous trials, including KEYNOTE-061 and KEYNOTE-062, have assessed the use of pembrolizumab alone and in combination with chemotherapy [19]. Pembrolizumab has less adverse effects than traditional chemotherapy, according to these trials, and it may occasionally result in long-lasting effects, particularly in individuals who exhibit elevated PD-L1 expression [19].

A total of 2,831 patients from four randomized controlled trials (RCTs) were included in this meta-analysis. Across all trials, the median follow-up duration was between 24 and 31 months. Pembrolizumab of 200 mg was administered to patients in the intervention group every three weeks for a maximum of two years, either by itself or in conjunction with chemotherapy. In contrast, the control group was given chemotherapy alone. The combined positive score (CPS), which takes into consideration the proportion of PD-L1-staining cells (tumor cells, macrophages, and lymphocytes) to all viable tumor cells, was uniformly used to quantify PD-L1 expression. Overall survival (OS), progression-free survival (PFS), and objective response rate (ORR) were all greater with pembrolizumab with chemotherapy than with chemotherapy alone in these trials. Comparing pembrolizumab monotherapy to chemotherapy, the former demonstrated superiority in OS and ORR but did not substantially improve PFS.

The results of our study offer well-supported recommendations about the long-term prognosis when choosing between pembrolizumab by itself or in combination with chemotherapy in clinical practice. Pembrolizumab by itself did not show any improvement in OS, according to our study [HR 0.87; 95% CI (0.76, 1.00), *p* = 0.04]. Chemotherapy was found to be superior to pembrolizumab, [HR 1.47; 95% CI (1.20, 1.81), *p* = 0.0002] but pembrolizumab in addition to chemotherapy had a significant advantage over chem. However, compared to patients receiving chemotherapy alone, those receiving pembrolizumab with chemotherapy had a significantly better overall survival rate [HR 0.80; 95% CI (0.72, 0.88), *p* < 0.00001]. In terms of PFS chemotherapy, [HR 0.78; 95% CI (0.70, 0.86), p, 0.00001]. For ORR, pembrolizumab monotherapy did not show statistically significant difference as compared with chemotherapy [RR: 0.68; 95% CI (0.30, 1.52), *p* = 0.34]. However, pembrolizumab and chemotherapy synergy showed better ORR compared with chemotherapy alone [RR 1.24; 95% CI (1.13, 1.36), *p* < 0.00001]. The synergistic effect of immunotherapy and chemotherapy, as well as the activation of neoantigen release brought on by chemotherapy, may be responsible for the significant improvement in ORR and PFS.

In terms of safety, the analysis revealed that pembrolizumab-based therapy (pembrolizumab alone or with chemotherapy) is associated with a greater frequency of adverse events (AEs) of any grade and severe (grade 3–5) than with chemotherapy alone. The corresponding hazard ratios (HRs) of 0.77 and 0.55 suggest more frequent and severe AEs in the pembrolizumab group. Despite this, there was no discernible variation in the rates of treatment termination, indicating that AEs may not necessarily lead to stopping therapy. For common side effects like fatigue, anorexia, and anemia, there were no significant differences between the two groups, but nausea was more frequent with pembrolizumab-based therapy (HR 0.60). The analysis highlights a significantly increased risk of unfavorable immune-related events (IRAEs) with pembrolizumab, particularly hypothyroidism (HR 3.83). This reflects pembrolizumab’s mechanism, which enhances immune responses but can also lead to immune-mediated toxicities [20]. Clinically, while pembrolizumab offers substantial cancer treatment benefits, these findings highlight the necessity of closely monitoring and controlling IRAEs to enable patients to successfully continue their treatment. Balancing the benefits and risks is crucial for optimizing patient outcomes.

According to the recent FDA Guidelines, only patients with these malignancies whose tumors also have elevated levels of another protein, PD-L1, are now eligible to receive pembrolizumab. PD-L1 levels are measured using a combined positive score (CPS), and patients whose tumors have a PD-L1 CPS of 1 are eligible for the updated approval or above [21]. In our meta-analysis, the subgroup analysis based on PD-L1 expression highlights that pembrolizumab’s efficacy is closely tied to PD-L1 levels. Pembrolizumab combo therapy did not improve overall survival (OS) in patients with PD-L1 CPS < 1 (HR 0.92), and data on pembrolizumab monotherapy were lacking for this group, suggesting minimal benefit for tumors with very low PD-L1 expression. However, for those with PD-L1 CPS ≥ 1, pembrolizumab in combination with chemotherapy significantly improved OS (HR 0.77), while monotherapy showed no benefit (HR 0.84). For patients with PD-L1 CPS ≥ 10, both pembrolizumab monotherapy and combination therapy provided significant OS benefits (HRs 0.69 and 0.71, respectively), indicating that higher PD-L1 expression strongly correlates with better outcomes. This implies that patients with high PD-L1 expression levels may benefit from pembrolizumab monotherapy, whereas those with lower PD-L1 expression levels may benefit more from combination therapy. Overall, these results emphasize the need for personalized treatment strategies based on PD-L1 expression to optimize pembrolizumab’s effectiveness.

By combining data from several studies, our systematic review and meta-analysis offer more thorough and trustworthy proof of pembrolizumab’s effectiveness. This makes our findings more broadly applicable, making them highly relevant for clinical decision-making. Unlike individual studies, our research makes it clear that combination therapy with chemotherapy greatly increases both overall survival (OS) and response rates, whereas pembrolizumab monotherapy provides only modest advantages for OS and progression-free survival (PFS). This distinction is critical for tailoring treatments to patient profiles. Additionally, we highlight the synergy between immunotherapy and chemotherapy, showing how it enhances clinical outcomes. Our study offers a more relevant and useful resource for optimizing treatment strategies based on a broader evidence base.

Despite the significant conclusions drawn from this systematic review and meta-analysis, it is crucial to note that there are certain limitations. First, long-term follow-up was absent from some of the investigations which restrict evaluating the safety and effectiveness of pembrolizumab in the long term. Finally, it is acknowledged that pembrolizumab has a synergistic effect with chemotherapy; however, the mechanism of this synergy remains unclear, which must be addressed in order to improve treatment algorithms.

## Conclusion

For advanced gastric cancer, this systematic review and meta-analysis assessed the safety and efficacy of pembrolizumab-based treatments. Our results demonstrate that in comparison with chemotherapy alone, pembrolizumab can considerably improve overall survival (OS) and progression-free survival (PFS), particularly when used in conjunction with chemotherapy. In patients with elevated levels of programmed death-ligand 1 (PD-L1), these advantages are especially noticeable.

While combining pembrolizumab with chemotherapy increases the chances of a positive response, it also comes with a greater risk of side effects, making careful monitoring essential to ensure patients maintain their quality of life.

In summary, our results support the integration of pembrolizumab into treatment plans for advanced gastric cancer, with a focus on strategies guided by patient biomarkers. Ongoing research is vital to further investigate the long-term safety and effectiveness of these therapies, ultimately aiming to improve outcomes for individuals facing this difficult diagnosis.

## Data Availability

The research data supporting the results of this manuscript were obtained from publicly accessible databases and previously published studies. These include randomized controlled trials (RCTs) sourced through PubMed, Scopus, Cochrane Library, and ClinicalTrials.gov as part of our systematic review and meta-analysis process. The protocol for this study is registered with PROSPERO (registration No. CRD42024572775) and is accessible at https://www.crd.york.ac.uk/prospero/display_record.php?ID = CRD42024572775. Data extracted from included RCTs are available in their respective publications. 1. Shitara K, Van Cutsem E, Bang YJ, et al. Efficacy and safety of pembrolizumab or pembrolizumab plus chemotherapy vs chemotherapy alone for patients with first-line, advanced gastric cancer: the KEYNOTE-062 phase 3 randomized clinical trial. JAMA Oncol. 2020;6(10):1571–1580. 2.Chung HC, Kang Y, Chen Z, et al. Pembrolizumab versus paclitaxel for previously treated advanced gastric or gastroesophageal junction cancer (KEYNOTE‐063): a randomized, open‐label, phase 3 trial in Asian patients. Cancer. 2022;128(5):995–1003. 3. Fuchs CS, Özgüroğlu M, Bang YJ, et al. Pembrolizumab versus paclitaxel for previously treated PD-L1-positive advanced gastric or gastroesophageal junction cancer: 2-year update of the randomized phase 3 KEYNOTE-061 trial. Gastric Cancer. 2022;25(1):197–206. 4. Rha SY, Oh DY, Yañez P, et al. Pembrolizumab plus chemotherapy versus placebo plus chemotherapy for HER2-negative advanced gastric cancer (KEYNOTE-859): a multicentre, randomised, double-blind, phase 3 trial. Lancet Oncol. 2023;24(11):1181–1195. 10.1016/S1470-2045(23)00515-6.
